# Case report: Dementia sensitivity to altitude changes and effective treatment with hyperbaric air and glutathione precursors

**DOI:** 10.3389/fneur.2024.1356662

**Published:** 2024-06-19

**Authors:** Edward F. Fogarty, Paul G. Harch

**Affiliations:** ^1^MoPlatte Sports Medicine, Ogallala, NE, United States; ^2^LSU Health Sciences Center, New Orleans, LA, United States

**Keywords:** hyperbaric, glutathione, pre-cursors, altitude, wellness, pandemic, dementia, COVID-19

## Abstract

A 78-year-old man with dementia experienced waxing and waning of symptoms with changes in altitude as he traveled from his home in the Rocky Mountains to lower elevations and back. To replicate the improvement in his symptoms with travel to lower elevations (higher pressure), the patient was treated with a near-identical repressurization in a hyperbaric chamber using compressed air. With four 1-h treatments at 1.3 Atmospheres Absolute (ATA) and concurrent administration of low-dose oral glutathione amino acid precursors, he recovered speech and showed improvement in activities of daily living. Regional broadcast media had documented his novel recovery. Nosocomial COVID-19 and withdrawal of hyperbaric air therapy led to patient demise 7 months after initiation of treatment. It is theorized that hyperbaric air therapy stimulated mitochondrial biochemical and physical changes, which led to clinical improvement.

## Introduction

The effects of barometric pressure on human physiology and cognition date back to Evangelista Torricelli's invention of the barometer in 1643. In 1644, Torricelli observed, “We live submerged at the bottom of an ocean of the element air.”(1) Altitude ascents and descents in air have an effect on human physiology due to changes in both oxygen pressure and barometric pressure. The positive effects of descent from altitude on human pulmonary, rheumatic, and cardiac diseases have been documented during relocations of people from the Rocky Mountains (5000–10,000 ft above sea level) to the lower Missouri River Basin of the United States (~1000 ft above sea level) (2, 3). Kramer et al. (4) quantified and showed the durability of this phenomenon in chronic obstructive pulmonary disease (COPD) patients who relocated for 3 weeks from Jerusalem (altitude 800 m, 2500 ft, 13.4 psi, 0.91 ATA) to the Dead Sea (altitude 402 m of air below sea level 15.4 psi, 1.05 ATA). At the end of the Dead Sea relocation, parameters such as walking distance and maximum oxygen consumption improved and persisted for 2 weeks after returning to Jerusalem at 0.91ATA (4). The findings by Kramer et al. were substantiated by 50 years of physiological evidence of the bioactivity induced by pressure changes in the same range as his COPD patients who relocated from Jerusalem to the Dead Sea, moving from 1.0015 ATA to 1.3 ATA (5).

The opposite effect, i.e., worsening of COPD on ascent in “the ocean of air,” was observed by Dr. Orval Cunningham, professor of anesthesiology (University of Kansas), the specialty of gas physiology. While on a vacation to the Colorado Rocky Mountains during the Spanish flu pandemic of 1918, he became aware of the increased mortality of Spanish flu victims at higher altitude (6). He reasoned that, since a descent to lower elevations has a therapeutic effect on patients (2, 3), a further increase in pressure beyond sea level, i.e., to below “the ocean of air,” would have further benefit. Using a converted boiler as a hyperbaric chamber in Kansas City, Missouri (altitude 909 ft), Cunningham replicated this improvement caused by altitude descent on human disease when he treated an agonal Spanish flu patient in 1918, achieving complete recovery with four daily 1-h 1.6 ATA hyperbaric air treatment (HBAT) sessions (6, 7) Countless Spanish flu patients followed, and Cunningham extended his clinical treatment benefits to a variety of conditions (7–9). After a hundred years, his reasoning and success were replicated in the application of hyperbaric oxygen therapy (HBOT) to COVID-19 (10–12)^.^ and post-COVID “Long Haulers Syndrome.” (13–15).

The results obtained by Kramer et al.^.^ (4) and Cunningham with compressed air (2, 3, 6–9) have been replicated in cerebral palsy children (16, 17), a drowned child (18), and two persistent postconcussion syndrome studies (19, 20). The pressure changes observed in these studies were in the range of the pressure changes observed in the Rocky Mountain/Great Plains relocators (2, 3) and the pressure changes experienced by our dementia patient on weekend trips from his home in the Rocky Mountains to western Nebraska. During these trips, he experienced clinical deteriorations and improvements that tracked altitude changes. Based on the above literature, the authors' personal experience, and the patient's clinical travel experience, the primary author replicated the clinical benefits experienced on the weekend trips to lower altitude (higher pressure) by treating his patient in a portable hyperbaric chamber. In this study, we report the sustained improvement in dementia with repetitive hyperbaric air therapy, using pressure similar to that used by others who have used altitude descent to improve their medical conditions (2–4).

## Case description

**Figure 1:**
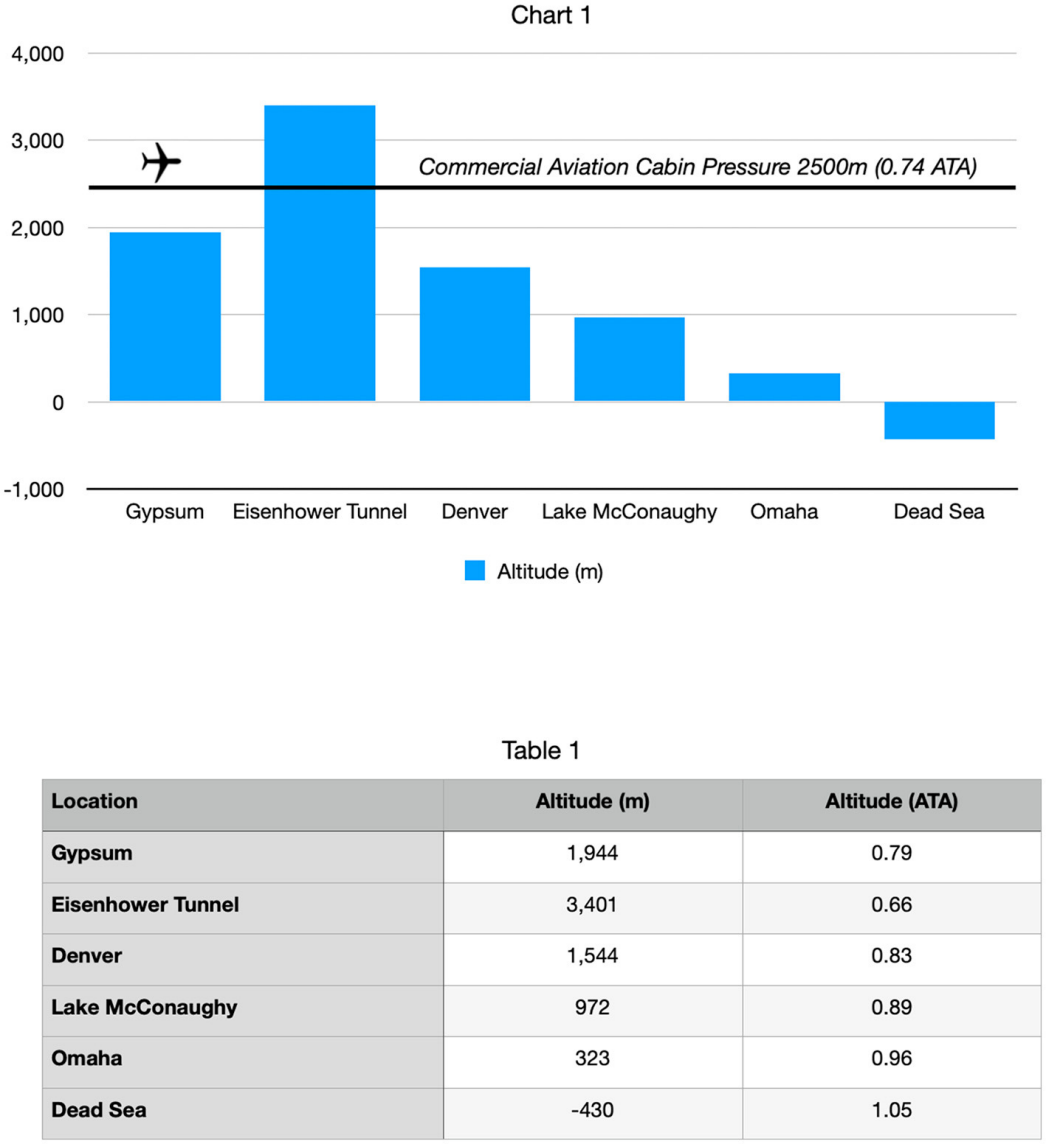
Schematic of relevant altitudes discussed in the case report.

The patient was a 78-year-old man with dementia who lived for 16 years in Gypsum, Colorado at 6400 feet (1944 m, 0.79 ATA, 11.6 psi) and was a summertime vacation home neighbor of the primary author in Lake McConaughy, Nebraska (3330 feet altitude, 972 m, 0.89 ATA, 13.0 psi). He was cognitively intact until a cerebral injury from an urgent cholecystectomy in the spring of 2019. Post-operatively, the family noticed an immediate cognitive change (“he was no longer himself”). The patient was reduced to social withdrawal, infrequent smiles, and little intelligible speech. He was subsequently diagnosed with dementia. In May 2019, his son-in-law attended a baseball game with the patient in Denver, Colorado (21) (altitude of 5200 feet, 1544 m, 0.83ATA, 12.1 psi) and noticed that he had become incoherent (delirious) around 4:00 PM during the middle innings of the game. Attendance at this game included travel from his home at an elevation of 6400 feet (0.79 ATA) over the Continental Divide (Eisenhower Tunnel, 11,111 feet, 3401 m, 0.66 ATA, 9.6 psi) and a pressure change of−0.13 ATA (see Figure 1). This was the first episode where the patient's sundowning had occurred well before its typical evening time.

Travel to the baseball game entailed 90 min of increasing hypobaric hypoxia from his home at 6400 ft (0.79 ATA) to the Eisenhower Tunnel (11,111 feet, 0.66 ATA) before descent to the baseball stadium at 5,280 ft (0.83 ATA). This included a peak stress of 15–20 min at the Eisenhower Tunnel. The early sundowning was confirmed by additional family members and the primary author's observations on multiple occasions during the summer of 2019 while traveling on the same route to his vacation home in Nebraska for weekend trips (3,330 ft., 0.89 ATA). After staying for a number of days at the decreased altitude (higher pressure), typically on the third day (Sunday afternoons), the family observed behavioral and symptomatic improvement. The primary author also witnessed the patient's cognitive deterioration post cholecystectomy, his further deterioration during visits of summer 2019, and rebounding by Sunday afternoons during vacation home visits.

The observations during the baseball game travel suggested that the hypobaric/hypoxic stress induced by road travel through Eisenhower Tunnel (0.66 ATA) on the patient's dementia (22, 23), a 0.13 ATA decrease in pressure and oxygen pressure, was similar to Cunningham's observations of Spanish flu patients in the Rocky Mountains (6). The lower-altitude higher-pressure rebound improvement observed in the mental status of the patients during Sunday afternoons, a 0.10 ATA increase from his hometown and a 0.23 ATA (3.4 psi) increase from the hypobaric/hypoxic stress zone of Eisenhower Tunnel (0.66 ATA), was in the range of bioactive pressure changes described by Kramer and Godfrey (4) (0.14 ATA increase in pressure), effects of altitude descent from the Rocky Mountains to the Great Plains (2, 3), recompression effects on patients with altitude sickness (24, 25), and the MacDonald–Fraser review of bioactivity in the micropressure range of 1.015–1.3 ATA (5). Based on the authors' previous low-pressure hyperbaric therapy experience with an Alzheimer's patient (26), a drowned child (18), and the medical literature (vide supra), the primary author proposed that the beneficial pressure changes experienced by the patient could be replicated in a portable chamber. Given the effects of HBOT on Nrf2 upregulation (27–29) and of NRF2 effects on intracellular glutathione (GSH) (30), supplemental GSH precursors were recommended adjunctively during hyperbaric treatment.

## Treatment

The patient was initiated on daily 1.6 g sublingual glutathione amino acid precursors 3 weeks prior to hyperbaric treatment. The primary author and his wife trained the patient's spouse, children, and adult grandchildren on chamber operations, and hyperbaric treatment was commenced on 20 October 2019, approximately 6 months after cholecystectomy-induced cerebral injury. The treatments were carried out once/daily, 5 days/week, at 1.09 ATA/45–60 min of total treatment time for 20 treatments, 1-week break, repeat 20 treatments. The 0.3 ATA increase in pressure in each hyperbaric treatment was the same increase in pressure used in hyperbaric air treatment of a sub-acutely drowned 2-year-old girl in which global regrowth of brain tissue was demonstrated (18).

### Outcome and follow-up

**Figure 2:**
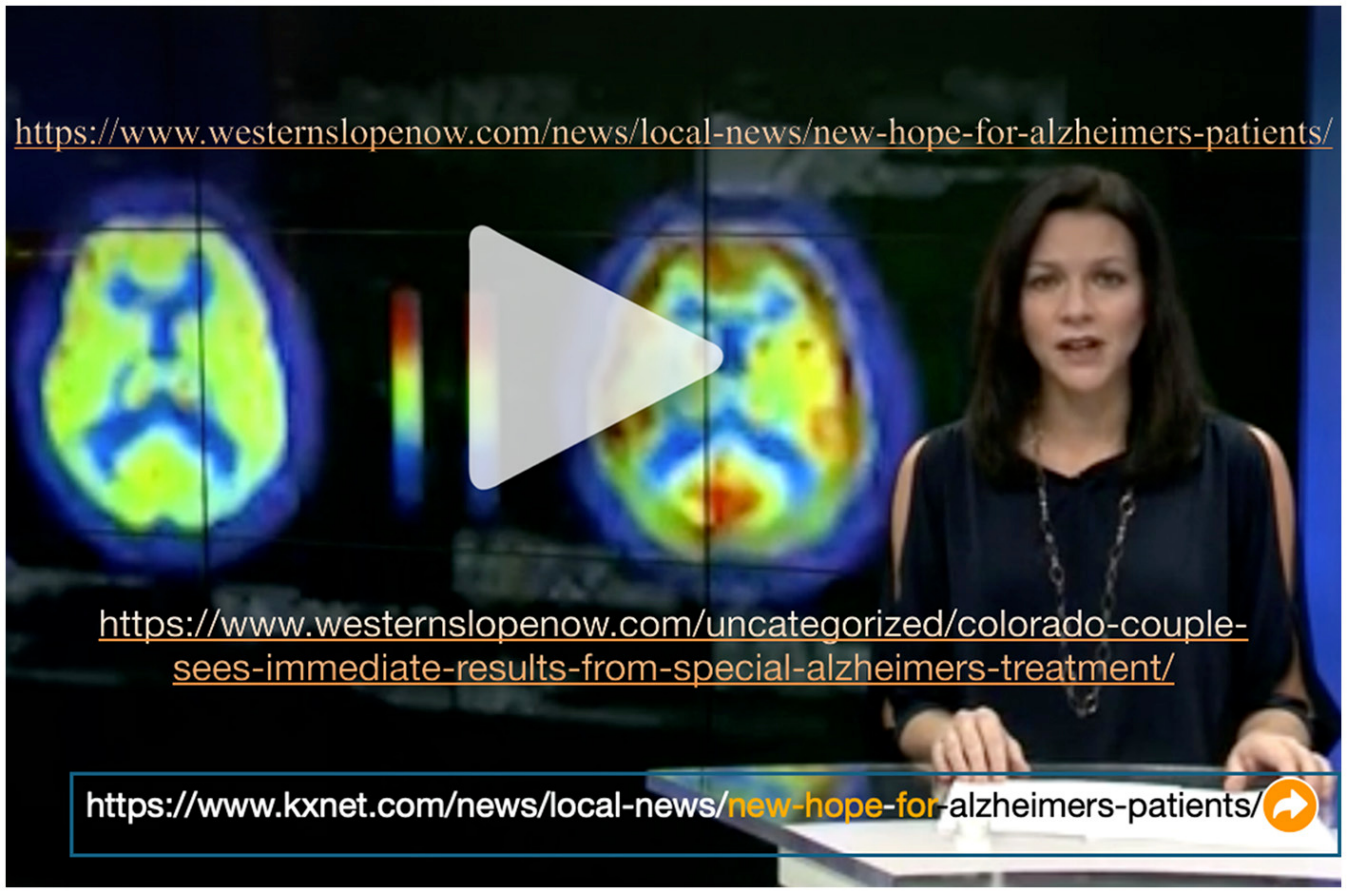
Colorado/North Dakota television reports links.

**Figure 3:**
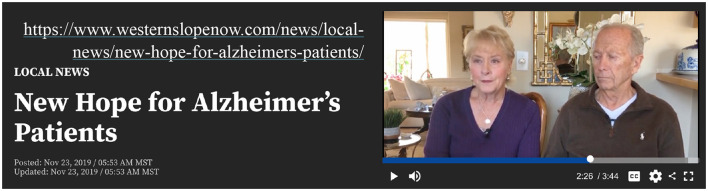
11/23/2019 Local media news report of the patient and his wife after 14 hyperbaric treatments. The patient recounts his improvements in activities of daily living. At the 2:26 timestamp of the feature, the patient's spouse relates her husband telling her on day 4 of the therapy, “that machine is working, it's helping me - I know it is.” The patient had little to no functional speech before the treatment.

The patient received 14 hyperbaric treatments in 20 days. After the first four treatments, the improvements in speech, cognition, and activities of daily living were so significant that two television news stations in two different US states reported the patient's progress in separate broadcasts. See broadcast news links shown in Figures 2, 3 and Supplementary material.

The broadcasts demonstrate the return of the patient's quick wit that had disappeared after the cholecystectomy and was still absent on the Sunday before the establishment of hyperbaric/glutathione therapy. The couple humorously referenced the matter of their upcoming 50th anniversary in March of 2020, with the patient stating that it should be remarkably different than what was expected before the establishment of the hyperbaric treatment.

Over the next 4 months, the patient received 67 HBATs at 0–5/week with 11.2 g of GSH precursors. He maintained his cognitive gains and re-engaged in exercising, carried out household chores, and performed activities of daily living until a hospitalization for a urinary tract infection led to him contracting COVID-19 (March, 2020). During the 2-month hospitalization, HBAT and GSH precursors were unavailable, and the patient experienced significant cognitive decline. He was discharged home on multidrug therapy due to worsened dementia/agitation in early May of 2020. He required significant in-home nursing support, was no longer speaking, was frequently combative, and was occasionally delirious, i.e., much worse clinically than when undergoing prehyperbaric therapy and GSH precursors 7 months before. The patient no longer remembered or understood the improvement he had experienced with hyperbaric treatment and refused all attempts at treatment, except for a 1-h session in early June 2020. That single hour of therapy caused an immediate calming of the patient, allowing a trip to the local grocery store later in the day (31). This was his first outing in over 3 months of institutional and home confinement.

Encouraged by the trip to the grocery store, the family traveled to their vacation home (0.89 ATA) the following weekend, incurring another hypobaric/hypoxic stress in the Eisenhower Tunnel (0.66 ATA). Once at the vacation home, the patient went missing, prompting a neighborhood manhunt that found him huddled in the crawl space under his home (31). The family returned to the Colorado home the following morning by the same route, incurring another hypobaric/hypoxic stress event through the Eisenhower Tunnel. Upon arrival, agitation worsened over the next several days, necessitating sedation. An extreme nocturnal delirious event occurred requiring physical restraint (88), and the patient expired during sleep within a few days.

## Discussion

This case report demonstrates the sensitivity of a patient's dementia to altitude ascents and descents and the replication of beneficial altitude descent conditions with hyperbaric chamber air treatments of the same magnitude. An increase in altitude is a hypobaric/hypoxic stress to living organisms (1, 25). Altitude hypobaric/hypoxic stress is a routine event with nearly every commercial airline flight. Because of in-flight cardiac arrests in patients with coronary artery disease, automatic external defibrillators have been placed in commercial airlines (32). Commercial airline-induced hypobaric/hypoxic stress has also been demonstrated to induce acute delirium in elderly cognitively impaired patients with neurodegenerative disease (33). In younger individuals, Ewing et al. (34) reported cognitive deficits induced by hypobaric/hypoxic stress on asymptomatic college students with a 1–3-year-old mild traumatic brain injury. A traumatic brain injury (TBI) group and a matched control group underwent cognitive testing at 12,467 feet (0.63 ATA) in a hypobaric chamber. Testing at 0.63 ATA demonstrated a statistically significant reduction in cognition in the TBI group compared to the matched controls, and this value was equivalent to acutely concussed patients.

The opposite change in pressure, a decrease in altitude/increase in pressure, is a hyperbaric hyperoxic recompression, similar to the treatment of mountain sickness (25) and divers with decompression illness (35). Duplicating the experience of demented patients in Sadlon et al.'s review (33), our patient repetitively experienced a deterioration in his dementia symptoms with intermittent trips to over 4,700 feet higher than his home elevation of 6,400 feet (Figure 1). These exacerbations of dementia symptoms were ameliorated by sustained descent to a lower elevation than his home, as reported in the literature (2–4). To improve his dementia symptoms, the same degree of pressure increase in his altitude descents was repetitively duplicated in a home-portable hyperbaric chamber. Each hyperbaric treatment was nearly equivalent to a virtual 1-h transport from his home altitude (0.79 ATA) in the Colorado Rockies to a theoretical landing in the Dead Sea (1.05 ATA), as described for the patients in the study by Krramer et al. (4). Fourteen repetitive hyperbaric air recompressions (“Dead Sea excursions), in combination with glutathione precursors to combat oxidative stress and inflammation, achieved marked cognitive improvement. The improvement was sustained for 4 months by additional hyperbaric and precursor therapy until the patient's demise from COVID-19 brain injury and repetitive hypobaric/hypoxic stress during altitude changes.

This case suggests the bioactivity of the unappreciated component of hyperbaric therapy, barometric pressure, due to the known similar effects in treatment of mountain sickness (25). HBOT has been traditionally defined as the use of 100% oxygen at > 1.4 ATA (35), and more recently > 1.0 ATA (36). These definitions ignored the effect of barometric pressure until the United States FDA pointed this out in 2011 (37). Recent gene expression experiments indicate that barometric pressure may be the dominant component of hyperbaric and hyperbaric oxygen therapy (38). In our patient, the recompression during motor vehicle travel to lower elevations caused equal increases in oxygen and barometric pressure, but the overall pressure of oxygen and amount of oxygen are a fraction of the typical pressures and amounts used in clinical hyperbaric oxygen therapy (36). Such small increases in oxygen and pressure have shown biological (5) and significant clinical effects for altitude sickness (25) but are not appreciated as a treatment for chronic neurological disease. In our case, the initial hyperbaric chamber treatments could not be construed as treating acute injury from the hypobaric/hypoxic trips to over 11,000 feet since our patient had been at his Gypsum home altitude (0.79 ATA) for 6 weeks prior to initiating hyperbaric treatment. The repetitive treatments appeared to duplicate the well-known trophic healing effects of repetitive hyperbaric therapy in other chronic conditions (36). The clinical improvements were durable until acute neurological injury from COVID-19 infection and repetitive hypobaric/hypoxic insults caused his death. His death mirrored the observation of altitude intolerance of chronic disease patients (2, 3, 6) and Dr. Cunnmingham's observation of increased mortality of Rocky Mountain dwelling patients with Spanish Flu (6), a disease very similar to COVID-19.

The precedence for our patient's improvement dates back to the 300-year initial history of hyperbaric therapy, which consisted solely of compressed air usage (6). This experience, long-forgotten by the modern hyperbaric oxygen therapy community, is undergoing a re-validation with accumulating animal and clinical studies documenting the bioactivity of lower pressures of compressed air, oxygen-enriched compressed air, and 100% oxygen in a variety of conditions (4, 16, 17, 19, 20, 39–43). Our patient's experience and this body of literature strongly indicate HBAT and low-pressure HBOT as therapeutic options for human disease.

The contribution of glutathione (GSH) amino acid precursors to our patient's recovery is impossible to prove but is suggested by the neuroprotective properties of GSH (44). GSH is an essential antioxidant against reactive oxygen and nitrogen species and is critical in regulating mitochondrial function, maintaining cell redox homeostasis, cell cycle regulation, apoptosis, immunological defense, and modulating pathological abnormalities (45, 46). Its deficiency is centrally involved in the pathophysiology of inflammation and many diseases, particularly Parkinson's disease, Alzheimer's disease, and amyotrophic lateral sclerosis (46–48). The glutathione amino acid formulation administered to our patient has been shown to increase intracellular glutathione (GSH) levels in cell cultures infected with RNA viruses (49).

Intracellular GSH levels are controlled by Nrf2 expression (30). Nrf2 regulates the cell's adaptive response to oxidants and electrophiles (30) and is increased (28, 29) and decreased (50) in diseases and hypobaric/hypoxic stress (51). Nrf2 upregulation has achieved disease amelioration in multiple conditions with multiple therapeutic agents (47, 52). This “systems medicine mechanism-based approach” to disease treatment (53) has described Nrf2 upregulation therapy as a “several diseases, one medicine” therapy; in summary, it is pleiotropic (47). Nrf2 is also upregulated by hyperbaric oxygen therapy in normal human endothelial cells (27) and in diseases (28, 29). We speculate that combining GSH precursors with HBAT caused a synergistic elevation of GSH, improvement of mitochondrial function through a mitohormesis effect, and clinical improvement in our patient. Upregulation of Nrf2 by hyperbaric therapy may explain the potentially unjustified criticism of widespread historical application to 132 diseases (54).

**Figure 4:**
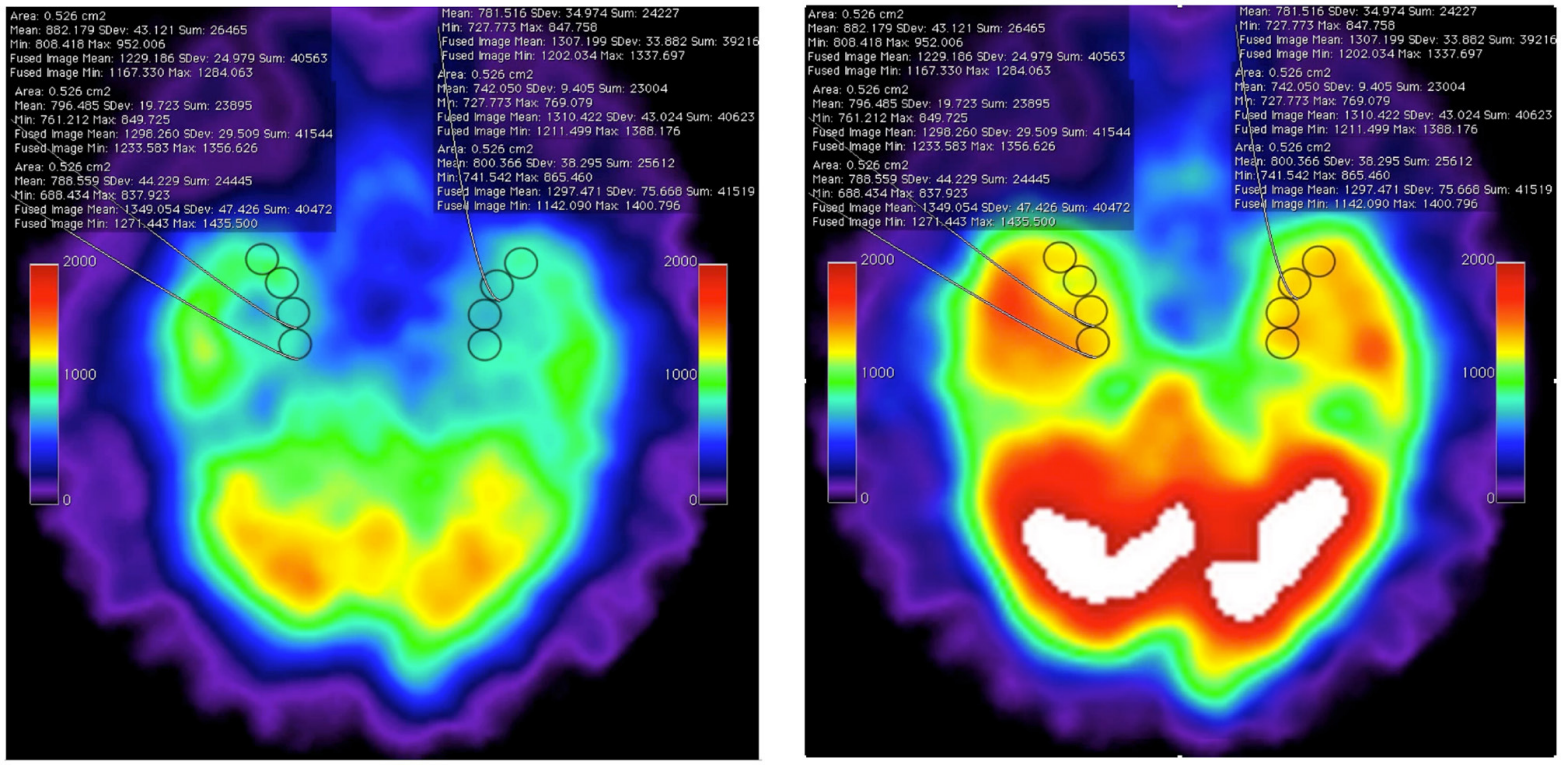
Axial SPECT imaging data from pre-HBOT baseline **(left)** and post 1-h HBOT data set **(right)** showing the inferior temporal lobes with eight regions of interest (ROI) in the medial temporal lobes. The statistical overlay documents increased intracellular GSH levels by Tc-99 HMPAO sequestration. These still images are captured from frame 1 and frame 20 of post-processed fusion of the two individual datasets performed on open-source Osirix clinical workstation software (see the source fusion video in Supplementary material). The “fused image” statistical data are obtained from the post-1-h HBOT HMPAO SPECT exam. Note that, at all ROIs, the uptake is increased over baseline by <30% [research data, Harch et al. (59)].

This case report did not include functional imaging documentation. In previous reports, we have documented HBOT-induced changes in chronic neurological disease with functional imaging changes in both SPECT (55–58) and PET (26). For example, the videographic fused images in Supplemental material and bookend still images of Figure 4 show improvement of function greater than 30% across the regions of interest obtained. The fused SPECT data set was obtained as the “prognosticator” data set of the whole brain after the first hour of HBOT at 1.5ATA. These images indirectly suggest the initial overexpression of Nrf2-generated GSH as stimulated by reactive oxygen and reactive nitrogen species (60). In the presented HBAT case, it is surmised that supplemental GSH precursors and hyperbaric air therapy contributed a greater percentage of reactive nitrogen species to the overall effect due to the 4:1 ratio of nitrogen to oxygen in air. SPECT in these cases may have been a proxy for Nrf2 upregulation. The intracellular trapping of the HMPAO SPECT radiopharmaceutical is mediated by glutathione levels in astrocytes (61). The HBOT SPECT brain blood flow and associated clinical improvements may have resulted, in part, from Nrf2-induced increases in astrocyte GSH. In the absence of HBAT and GSH precursors, we further speculate that the patient's cognitive decline post COVID-19 was due to a decline in intracellular glutathione. Glutathione depletion has been suggested as a major cause of the morbidity and mortality of COVID-19 (62).

## Conclusion

Cognitive deteriorations and improvements occurred in an elderly dementia patient with hypobaric/hypoxic motor vehicle ascents and descents from altitude. Temporary beneficial motor vehicle descents from altitude were replicated in a portable hyperbaric chamber. Fourteen hyperbaric treatments and oral glutathione precursor supplements resulted in cognitive improvements that were documented in television news mini-documentaries. The improvements were sustained for 4 months with additional hyperbaric and supplement treatment until the patient's hospital admission for UTIs, leading to nosocomial COVID-19 infection. Patient death was due to long COVID-19 dementia exacerbated by repetitive altitude insults. Hyperbaric-induced upregulation of NRF2-mediated glutathione synthesis is suggested as a contributory mechanism of action.

## Data availability statement

The datasets presented in this article are not readily available because of ethical and privacy restrictions. Requests to access the datasets should be directed to the corresponding author.

## Ethics statement

Ethical review and approval was not required for the study on human participants in accordance with the local legislation and institutional requirements. Written informed consent from the patients/participants or patients/participants' legal guardian/next of kin was not required to participate in this study in accordance with the national legislation and the institutional requirements. Written informed consent was obtained from the individual(s) for the publication of any potentially identifiable images or data included in this article. Written informed consent was obtained from the participant/patient(s) for the publication of this case report.

## Author contributions

EF: Conceptualization, Data curation, Formal analysis, Funding acquisition, Investigation, Methodology, Project administration, Resources, Software, Supervision, Validation, Visualization, Writing – original draft, Writing – review & editing. PH: Data curation, Methodology, Conceptualization, Formal analysis, Project administration, Investigation, Writing – review & editing.
