# Supplementary material for: Case report: Dementia sensitivity to altitude changes and effective treatment with hyperbaric air and glutathione precursors
Source: Front Neurol. 2024 Jun 19;15:1356662. doi: 10.3389/fneur.2024.1356662 (PMC11229546; doi:10.3389/fneur.2024.1356662)
Supplement: Supplementary file 1 [file Presentation_1.pdf]

Supplementary Materials: Presentation 1 Media Link

Additional media presentation (see link or click here) of patient and spousal experience with altitude wellness HBAT protocols in fall of 2019 from KXNET/Western North Dakota media:

https://www.kxnet.com/news/local-news/new-hope-for-alzheimers-patients/
